# A Case of Atrial Tachycardia Circulating around a Left Atrial Roof Scar with Diabetes Mellitus and Renal Failure on Hemodialysis

**DOI:** 10.1155/2016/6485939

**Published:** 2016-04-11

**Authors:** Naoko Hijioka, Masashi Kamioka, Hitoshi Suzuki, Yasuchika Takeishi

**Affiliations:** Department of Cardiology and Hematology, Fukushima Medical University, Fukushima 960-1295, Japan

## Abstract

*Introduction*. Little is known about the effects of volume change by hemodialysis (HD) and mechanical stress caused by an anatomical structure being in contact with the left atrium on the progression of atrial remodeling. We experienced a case of atrial tachycardia (AT) in a patient who had left atrial (LA) scarring at the LA roof and a low-voltage area with slow conduction around the LA scar as components of AT circuit. Here, we present the conceivable hypothesis of the LA scar and the low-voltage area formation. Our concept can be useful in developing a strategy for ablation in a patient with chronic renal failure (CRF) on HD.* Case Report*. A 65-year-old man with CRF on HD was referred for AT ablation. Three-dimensional electroanatomical mapping revealed that the AT conducted around an LA scar in a counterclockwise fashion. There was a slow conduction area at the superior side of the LA scar, where the AT was terminated during the ablation. Computed tomography indicated a close relationship between the LA and the anatomical structures (ascending aorta and pulmonary artery).* Conclusion*. Volume change by HD and close contact of anatomical structures to the LA can promote atrial remodeling, resulting in AT occurrence.

## 1. Introduction

It is well known that diabetes mellitus (DM) and chronic renal failure (CRF) promote the remodeling of atrial muscles [1, 2]. In contrast, there are limited data on how mechanical stress due to hemodialysis (HD) influences atrial remodeling [3]. We experienced a case of atrial tachycardia (AT) circulating around a left atrial (LA) roof scar in a DM and CRF patient on HD. Echocardiography showed a remarkable change in LA diameter before and after HD. Three-dimensional (3D) electroanatomical mapping merged with computed tomography revealed that the LA scar was close to the pulmonary artery (PA), and the low-voltage area was close to the ascending aorta (AS-Ao). In the current case report, we present a hypothesis that short-term volume change by HD and mechanical stress by PA and AS-Ao can induce atrial remodeling, resulting in the formation of an AT circuit.

## 2. Case Report

**Figure 1:**
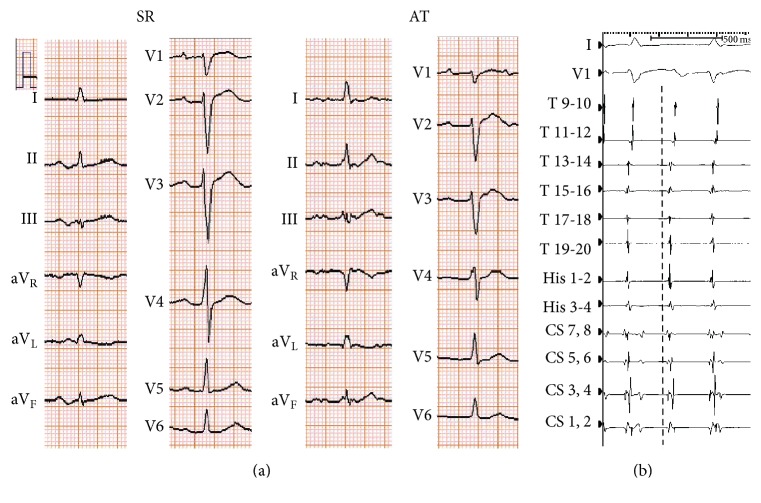
(a) The left panel shows a 12-lead electrocardiogram (ECG) in sinus rhythm (SR) and the right panel shows that of atrial tachycardia (AT). (b) Intracardiac ECG during AT. T indicates the Halo catheter. Numbers 9-10 are the distal electrodes located at the lateral tricuspid annulus (TA). Numbers 19-20 are located at the anteroseptal TA. His 1-2 is the distal electrode of His. CS indicates the electrode catheter in the coronary sinus (CS). CS 1-2 is the distal electrode and CS 7-8 is located at the CS ostium. The dotted line indicates the earliest activity of AT recorded at CS 7-8.

**Figure 2:**
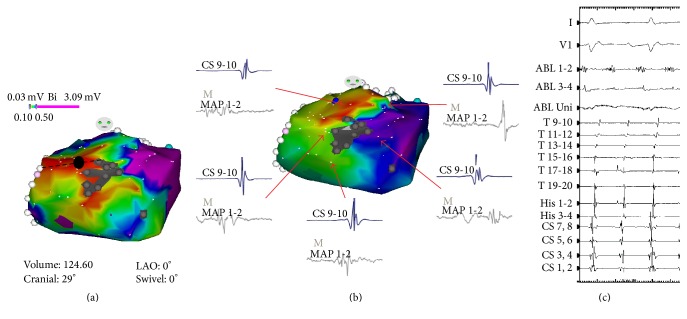
(a) The left atrial (LA) voltage map using a 3D electroanatomical mapping system. The grey area located at the anterior roof of the LA indicates the scar. The black circle indicates the site of entrainment pacing. The dotted line shows the linear ablation line between the LA scar and the ostium of the right upper pulmonary vein. (b) The LA activation map during AT. The arrowhead indicates local activity recorded around the scar. (c) Intracardiac ECG of the AT termination site recoded at the LA roof. ABL 1-2 indicates the distal electrode of the ablation catheter.

**Figure 3:**
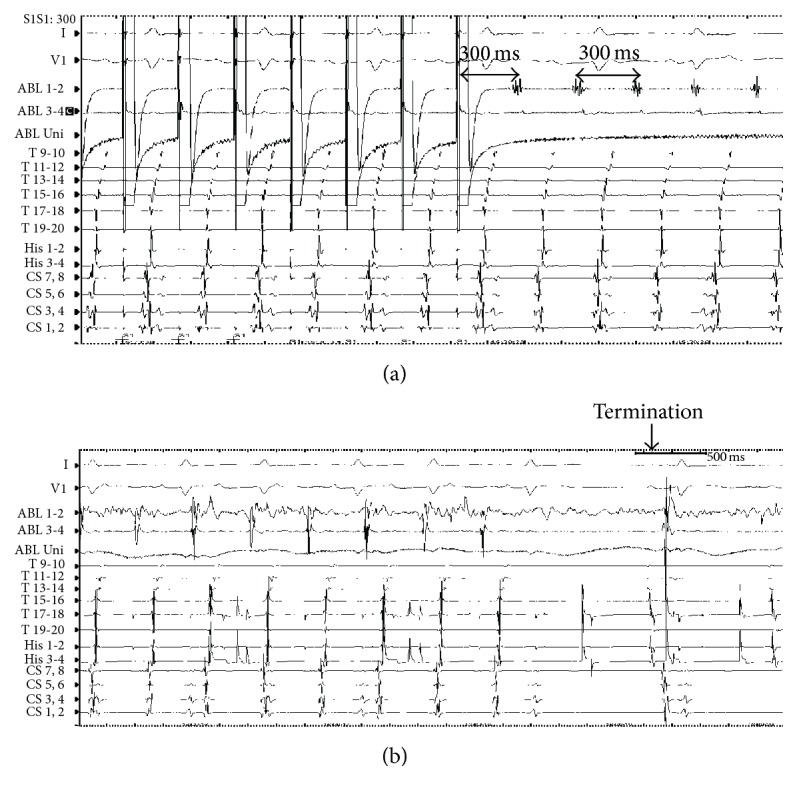
(a) Entrainment pacing at the LA roof with a stimulation interval of 290 ms slow conduction area as shown in Figure 2(a). The postpacing interval was the same as the AT cycle length. (b) The AT termination during the linear ablation.

A 65-year-old man was referred to our hospital for ablation of the AT. He had been treated for end-stage renal disease (estimated glomerular filtration rate: 9 mL/min/1.73 m^2^; serum sodium level: 139 mEq/L; serum potassium level: 3.6 mEq/L) due to DM and was receiving HD treatment. He felt palpitations and was diagnosed as having AT with a 12-lead electrocardiogram (ECG). Pilsicainide and amiodarone were prescribed; however neither medication could suppress the AT. Ablation was therefore considered. The patient's blood pressure (BP) was within normal range and his DM was well controlled (BP 110/70 mmHg and HbA1c 6.1%). Echocardiography showed normal left ventricular (LV) systolic function (ejection fraction: 56.5%) without LV dilatation (LV end-diastolic diameter: 52 mm). The LA was dilated (50 mm) prior to HD. The ECG recorded on admission showed AT with 290 ms PP intervals. P wave morphology was bimodal, positive, and wide in leads I, II, III, and aV_F_ and positive/negative in lead V1 (Figure 1(a)). The sinus rhythm ECG showed a different P wave morphology; lead I was flat, leads II, III, and aV_F_ were positive, and lead V1 was positive/negative (Figure 1(a)). An electrophysiological study was performed with a twenty-polar electrode catheter (Halo catheter) positioned along the tricuspid annulus (TA) and two octapolar electrode catheters positioned in the coronary sinus (CS) and at the bundle of His. The earliest local activity was recorded at the CS ostium at this time (Figure 1(b)). The total interval of atrial activity including TA, CS, and His electrodes did not fulfill the clinical AT cycle length (CL). We therefore advanced a mapping catheter into the distal CS, where a fragmented long duration potential was observed. After a transseptal puncture, 3D electroanatomical mapping of the LA was constructed using the CARTO system. In the LA voltage map, there was a scar area at the LA anterior roof. Around the scar, there were low-voltage areas with fragmentation at the roof and right anterior wall of the LA (Figure 2(a)). The activation map fulfilled almost the entire duration of the AT CL. This result indicated that the AT circulated around the scar in a counterclockwise fashion (Figure 2(b)). Entrainment pacing was performed at the LA roof (black circle in Figure 2(a)), where a fragmented potential was recorded (Figure 2(c)). The postpacing interval was in accordance with the AT CL (Figure 3(a)). Therefore, linear ablation was performed between the roof scar and the ostium of the right upper pulmonary vein (Figure 2(a)), during which the AT was finally terminated (Figure 3(b)). After completion of the ablation line, the AT could not be induced. Six months after ablation, no AT recurrence was observed.

## 3. Discussion

To the best of our knowledge, this is the first reported case of AT conducted around the scar at the LA anterior roof in a patient with DM on HD. There are two interesting points of observation; the first is the relationship between the P wave morphology of the AT and the conducting manner to the RA and LA. The second noteworthy observation is the anticipated mechanism of the scar and low-voltage area formation in the LA in a CRF patient on HD who had no structural heart disease or no history of AF.

**Figure 4:**
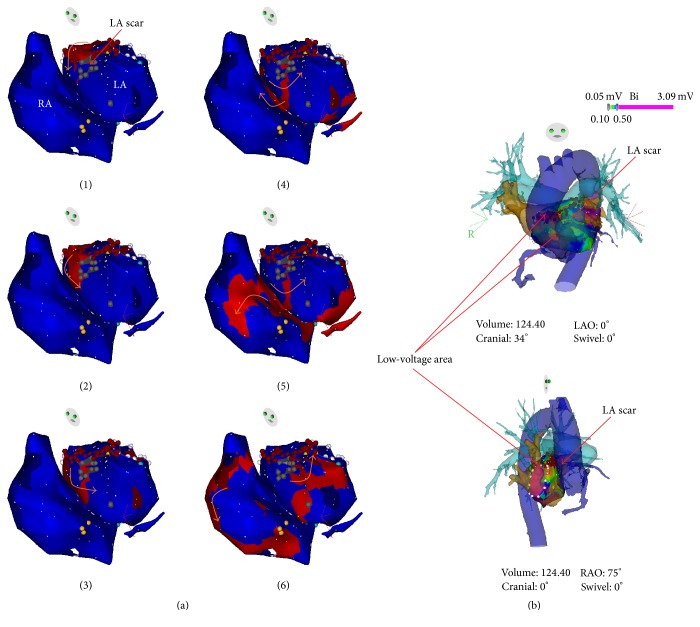
(a) The propagation map of AT with the LA, right atrium (RA), and CS. Arabic numbers indicate the time course of AT. The wavefront conducts around the LA scar with no excitation of the RA (images 1–3). The mid to inferior part of the RA began to activate (image 4). The RA wavefront went up to the RA septum and turned around the TA in a counterclockwise manner (images 5 and 6). The orange arrowhead indicates the AT wavefront. (b) The anatomical relationship between the LA low-voltage area and the ascending aorta, as well as between the LA scar and the pulmonary artery presented with the 3D electroanatomical map of the LA merged with computed tomography. As indicated by the red line, the LA scar was represented by grey area and the low-voltage areas were represented by red, yellow, and green areas.

The P wave morphology of the AT in the present case was positive and bimodal in leads I, II, III, and aV_F_ and positive/negative in lead V1. As shown in Figure 2(b), there were slow conduction areas in the right and the superior sides of the LA scar. The wavefront went down to the right side of the scar and turned left below the scar through the slow conduction area. This propagation manner constituted the first positive component of the P wave in leads I, II, III, and aV_F_. Interestingly, the AT wavefront propagated from the LA to the RA through the mid to inferior part of the septum only (Figure 4(a)), probably because of the Bachman bundle conduction disorder, which was generated by the existence of the anterior roof scar in the LA. The scar might have caused severe damage to the conductivity of the Bachman bundle. Hence, the wavefront, which propagated to the RA, went up the septum of the RA and turned around the TA in a counterclockwise manner, as shown in Figure 3(a). This is composed of the second positive component in leads I, II, III, and aV_F_ with a short duration of 50 msec. The duration of the P wave was about 190 msec and the residual duration of the AT CL was given by the slow conduction area located at the superior side of the LA scar (Figure 2(c)). The positive/negative P wave in lead V1 was explained by the spreading manner of the AT, which propagated from the mid to inferior septum of the LA to RA. This propagation style was similar to that of the AT originating from the aortic sinus of Valsalva. Wang et al. reported that the P wave morphology of AT originating from the noncoronary cusp and left coronary cusp is positive/negative [4].

Although it is well known that HD strongly promotes exacerbation of atherosclerosis, to the best of our knowledge, it is still unclear how LA remodeling progresses in patients with CRF on HD. Hori et al. reported that the contact areas, where the LA wall is anatomically in contact with the vertebrae, AS-Ao, or descending aorta (DS-Ao), are strongly associated with low-voltage areas and fractionated potentials [5]. Anatomical structures, such as the sinus of Valsalva, AS-Ao, DS-Ao, and the vertebrae, might put direct pressure on the LA and possibly extend the low-voltage area and the fragmentation potential formation, resulting in LA remodeling. In the present case, as shown in Figure 2(a), there was a scar area on the anterior superior of the LA. Low-voltage areas were found around the scar, specifically at the superior side (LA roof) and the right side of the scar (close to the RSPV). Notably, the scar region was located where a PA located in front of the scar with a short relative distance (Figure 4(b)). In addition, the low-voltage areas were very close to the sinus of Valsalva and the AS-Ao. Tekce et al. reported that even a single session of dialysis changed the LA diameter from 43.7 ± 5.3 mm (before HD) to 39.7 ± 4.6 mm (after HD) [6]. This result means that the stretching of the atrial myocytes occurred each time HD was performed, and the stretch itself has been proven to promote the LA remodeling. In the present case, the LA diameter measured by echocardiography also changed from 50.0 mm before HD to 45.7 mm after HD. Moreover, a dilated LA before HD might be considered to be closer to the PA and AS-Ao than that after HD. These areas of the LA were not only stretched by inner pressure before HD, but also compressed by anatomical structures (PA and AS-Ao). Such stretching and compression forces might injure the atrial myocytes and consequently induce a low-voltage area or scar formation in the LA.

## 4. Conclusion

LA volume changes caused by HD can create stretching forces in the atrium. The anatomical structure around the LA can provide the atrial myocytes with the compression force. Such mechanical stress might promote the LA remodeling, resulting in scarring or a low-voltage area, as well as a slow conduction area, formation in the absence of AF. This concept could help us develop a strategy for catheter ablation in CRF patients on HD in a more effective manner.
